# Ex-Vivo Stimulation of Adipose Stem Cells by Growth Factors and Fibrin-Hydrogel Assisted Delivery Strategies for Treating Nerve Gap-Injuries

**DOI:** 10.3390/bioengineering7020042

**Published:** 2020-05-05

**Authors:** Katharina M. Prautsch, Lucas Degrugillier, Dirk J. Schaefer, Raphael Guzman, Daniel F. Kalbermatten, Srinivas Madduri

**Affiliations:** 1 Department of Plastic, Reconstructive, Aesthetic and Hand Surgery, University Hospital Basel, University of Basel, Spitalstrasse 21, 4021 Basel, Switzerland; katharina.prautsch@unibas.ch (K.M.P.); LucasMichelJean.Degrugillier@usb.ch (L.D.); Dirk.Schaefer@usb.ch (D.J.S.); Daniel.Kalbermatten@usb.ch (D.F.K.); 2 Department of Pathology, University Hospital Basel, Hebelstrasse 20, 4021 Basel, Switzerland; 3 Department of Biomedical Engineering, University of Basel, Gewerbestrasse 14, 4123 Allschwil, Switzerland; 4 Department of Biomedicine, University Hospital Basel, Hebelstrasse 20, 4021 Basel, Switzerland; 5 Department of Neurosurgery, University Hospital Basel, Spitalstrasse 21, 4021 Basel, Switzerland; Raphael.Guzman@usb.ch

**Keywords:** adipose stem cells, neurotrophic factors, growth factors, peripheral nerve injuries, fibrin nerve conduits, hydrogels, stem cells delivery, axonal regeneration, Schwann cells

## Abstract

Peripheral nerve injuries often result in lifelong disabilities despite advanced surgical interventions, indicating the urgent clinical need for effective therapies. In order to improve the potency of adipose-derived stem cells (ASC) for nerve regeneration, the present study focused primarily on ex-vivo stimulation of ASC by using growth factors, i.e., nerve growth factor (NGF) or vascular endothelial growth factor (VEGF) and secondly on fibrin-hydrogel nerve conduits (FNC) assisted ASC delivery strategies, i.e., intramural vs. intraluminal loading. ASC were stimulated by NGF or VEGF for 3 days and the resulting secretome was subsequently evaluated in an in vitro axonal outgrowth assay. For the animal study, a 10 mm sciatic nerve gap-injury was created in rats and reconstructed using FNC loaded with ASC. Secretome derived from NGF-stimulated ASC promoted significant axonal outgrowth from the DRG-explants in comparison to all other conditions. Thus, NGF-stimulated ASC were further investigated in animals and found to enhance early nerve regeneration as evidenced by the increased number of β-Tubulin III+ axons. Notably, FNC assisted intramural delivery enabled the improvement of ASC’s therapeutic efficacy in comparison to the intraluminal delivery system. Thus, ex-vivo stimulation of ASC by NGF and FNC assisted intramural delivery may offer new options for developing effective therapies.

## 1. Introduction

Peripheral nerve injuries often result in loss of sensory and motor functions due to lack of effective therapeutic strategies, thus there is a great clinical need for developing new therapies [1,2]. Schwann cells (SC) play a crucial role in neuronal survival, axonal regeneration and re-myelination [3,4] by secreting an array of molecular signals, neurotrophic factors (NTF) and extracellular matrix proteins [5]. Furthermore, SC generate bands of Büngners for topographical guidance and path finding of the regenerating axons [6]. However, therapeutic use of SC is hampered due to the problems associated with harvesting the cells from healthy nerves and resulting co-morbidities [7]. Therefore, the need for stem cell-based therapies emerged for treating nerve injuries. 

Therapeutic stem cells should be easily accessible and undergo rapid proliferation and differentiation in vitro under controlled conditions. Mesenchymal stem cells (MSC) can differentiate into SC-like cells under specific stimuli and enhance peripheral nerve regeneration [8,9,10,11]. Not limiting to the source of bone marrow, MSC with multi lineage capacity can also be obtained from adipose tissue, dental pulp, umbilical cord blood and Wharton’s jelly of umbilical cord [12,13,14,15]. Adipose-derived stem cells (ASC) are easily accessible in large quantities from fat-tissue enabled by liposuction or abdominoplasty [6,13,16]. ASC proliferate rapidly [6] and possess a multi-lineage capacity, i.e., adipocytes, osteoblasts, chondrocytes [13,17,18]. Furthermore, ASC retain their mesenchymal potency over long-term culture [6,18] and promote the nerve regeneration similar to bone marrow stem cells [7]. In allogeneic transplantation, usage of ASC, like any other adult stem cells, benefits from their hypo-immunogenicity or immune-privilege by virtue of the reduced expression of HLA-DR class II histocompatibility antigen [19,20]. Thus, ASC may enable the development of “off-the-shelf therapies” for promoting the nerve regeneration. Within this context, neurotrophic potency of ASC and their delivery approaches play a crucial role in improving therapeutic efficacy.

As shown by many studies, factors that are present in the secretome of ASC promote axonal regeneration both in vitro and in vivo. When co-cultured with motor neuron-like cells or dorsal root ganglion (DRG) sensory neurons, ASC enhanced the neurite number and outgrowth length [21,22,23]. In line with these findings, several animal studies reported enhanced axonal regeneration and sensory-motor nerve conduction, when ASC were transplanted in polymeric nerve conduits (NC) or fibrin-hydrogel nerve conduits (FNC) [24,25,26]. Improved axonal growth and elongation was found in the FNC loaded with ASC in comparison to empty FNC [24,27,28]. However, an optimal delivery route for ASC still remains to be established in the context of peripheral nerve reconstruction. Various studies involving ASC therapy employed different cell delivery routes, i.e., injection into the lumen, dispersion within the fibrin conduit wall, dispersion within the lumen of fibrin matrix and coating on the luminal surface [7,22,26,27,29,30]. However, the therapeutic efficacy of the cells was scarcely correlated with delivery routes and carrier matrix in the context of nerve regeneration. Thus, the ineffective and incomplete outcome achieved so far by using the ASC therapy can be attributed largely to various structural and biochemical microenvironments that were inadequately orchestrated through different delivery routes. Moreover, studies focusing on the cell delivery strategies, for understanding the spatiotemporal influence of carrier matrix on the efficacy of therapeutic cells, are largely missing. Therefore, there is a clear need to investigate systematically the impact of important local delivery strategies on nerve regeneration and to establish effective options.

The most important soluble factors known to support the neuronal survival and axonal regeneration following traumatic nerve injury are NTF [4,31,32,33]. Nerve growth factor (NGF), amongst other NTF, specifically promotes survival and regeneration of sensory neurons by binding to the high-affinity trk-A receptors [34]. Vascular endothelial growth factor (VEGF) on the other hand is a potent angiogenic factor, which promotes proliferation of endothelial cells, formation and permeability of vascular structures [35]. Nevertheless, several studies demonstrated VEGF for having neurotrophic activities that were mediated either by flk-1 and flt-1 receptor binding or by enhanced vascularization [35,36,37,38,39]. 

Same as for ASC, various studies reported the improved nerve regeneration supported by exogenously administered NTF [31,34,40,41]. Despite these findings, the growth-promoting effects of ASC in response to a specific growth factors’ stimulus largely remain elusive in the context of nerve regeneration. On the other hand, rapid upregulation of NGF and VEGF after traumatic nerve injury [4,33] clearly shows the clinical significance for exposing the therapeutic impact of ASC following growth factors’ stimulation in the context of axonal regeneration.

We hypothesize improvement in the neurotrophic capacity of ASC in response to exogenous growth factors stimulation. Furthermore, a novel design of the fibrin hydrogel nerve conduits would facilitate discrete options for cell delivery and for enhancement of their therapeutic efficacy. Thus, the present study evaluated the capacity of ASC in response to specific stimuli of NGF or VEGF for promoting the axonal regeneration in vitro and in vivo. Furthermore, the influence of the stem cell delivery route, i.e., FNC assisted intramural vs. intraluminal ASC loading on early nerve regeneration was also investigated in rats by using a 10 mm sciatic nerve gap-injury model.

## 2. Materials and Methods 

### 2.1. Isolation and Culture of Adipose Stem Cells (ASC) 

All the in vitro studies were conducted in accordance with the local veterinary commission in Basel, Switzerland (No. 2925). Visceral adipose tissue was harvested from adult Sprague-Dawley rats and processed under sterile conditions as described earlier [21]. Briefly, the fat tissue was rinsed in 0.01 M phosphate-buffered solution (PBS), minced and resulting tissue was digested with 0.15% (w/v) Type I Collagenase (Gibco Life Technologies, Cat. No. 17100017) for 1 h at 37 °C and centrifuged for 5 min at 1500 rpm and 4 °C. The pellet was re-suspended in growth medium (GM) i.e., Dulbecco`s Modified Eagle`s Medium (DMEM, Gibco, Cat. No. 41965039) supplemented with 10% Foetal Bovine Serum (PAN-Biotech, EU-approved, Cat. No. P40-47500) and 1% Penicillin/Streptomycin (BioConcept, Cat. No. 4-01F00-H). Isolated ASC were seeded at a density of 3000 cells/cm^2^ and expanded at 37 °C with 5% CO_2_ in a humid atmosphere; GM was changed every 72 h. Cells were passaged using 0.25% Trypsin-EDTA (BioConcept, Cat. No. 5-51F00-H) at 90% confluence and resulting cells at passage 2 (P2) or 3 (P3) were used for the experiments.

### 2.2. ASC Characterization

Rat ASC (P2) were seeded on 24 well plates for characterization. Cells were fixed in 4% paraformaldehyde (PFA) at room temperature (RT) for 10 min and permeabilized and blocked in 1% normal goat serum (NGS) in PBS (i.e., dilution buffer) for 60 min at RT. ASCs were incubated overnight at 4 °C with the human mesenchymal stromal cell markers monoclonal mouse anti-CD44 (1:1000), monoclonal mouse anti-CD90 (1:200), monoclonal mouse anti-CD105 (1:200) and monoclonal rabbit anti-CD29 (1:100), and the hematopoietic marker polyclonal rabbit anti-CD45 (1:500) (Abcam, Cat. No. ab93758). Cells were then washed in PBS and incubated for 60 min at RT with the secondary antibody goat anti-mouse Alexa Fluor 488 (1:500, Abcam, Cat. No. ab150109) and goat anti-rabbit Alexa Fluor 488 (1:500, Abcam, Cat. No. ab150061) and Hoechst 33258 nuclear staining (1:1000, Sigma Aldrich, Cat. No. 94403). Subsequently, digital images were acquired at 20× magnification (numerical aperture 0.45) by using a Nikon Eclipse Ti2 fluorescent inverted microscope (Nikon Eclipse Ti2-E, -E/B, Nikon Corporation, Japan) and a Photometrics prime 95B 25 mm camera (Teledyne photometrics, Tucson, AZ, USA). The images were automatically stitched by the Nikon NIS-Elements AR image analysis software (NIS- Elements AR Analysis 5.11.00 64-bit, Laboratory Imaging, spol. s.r.o., Praha, Czech Republic). Furthermore, immunostaining images of ASC for various markers were analyzed quantitatively using ImageJ.

### 2.3. Isolation of Chicken Embryonic Dorsal Root Ganglions (DRG)

Fertilized chicken eggs were obtained from Gepro Geflügelzucht AG (Flawil, Switzerland). The eggs were shipped at ambient temperature and incubated at 37.8 ± 0.2 °C under 100% relative humidity for 10 days (E10). After incubation, the eggs were cleaned with 70% ethanol and opened under a laminar airflow cabinet to collect the embryos. E10 embryos were dissected following a standard dissection protocol under a stereomicroscope [34]. DRG-explants were collected from the lumbar part of the spine and transferred to GM for cell culture.

### 2.4. ASC Stimulation and Secretome Harvest 

ASC were seeded on 24 well plates at a density of 13,000/cm^2^ and 500 μL of GM was added with or without supplementation of exogenous growth factors, i.e., 10 ng/mL of recombinant human NGF or recombinant mice VEGF, as indicated in the experimental design. NGF (Cat. No. 256-GF) and VEGF (Cat. No. 493-MV) were obtained from R&D Systems (Minneapolis, MN, USA). Cells were cultured for 72 h and no growth medium was exchanged during culture to enrich the secretome. After 72 h of enrichment, the resulting conditioned medium enriched with secretome (STM) was collected and used for subsequent experiments.

### 2.5. Experimental Design In Vitro

**Figure 1:**
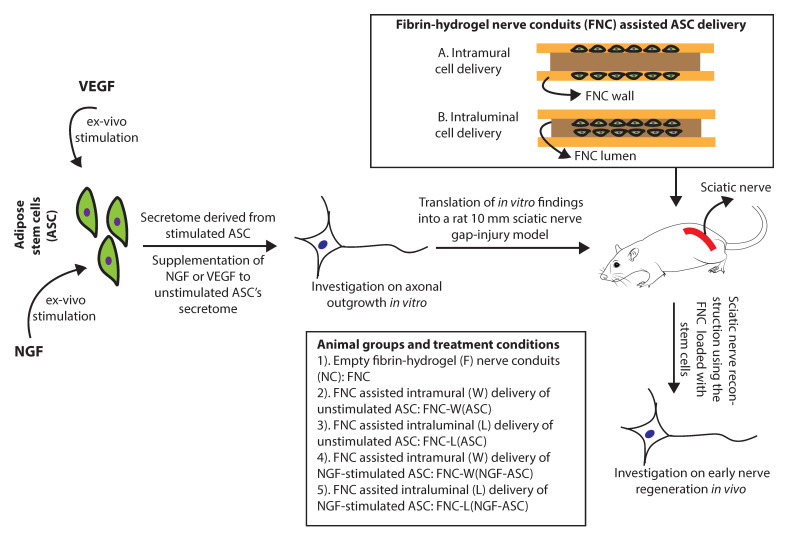
Ex-vivo stimulation of adipose stem cells using growth factors and fibrin-hydrogel nerve conduits assisted stem cell delivery strategies for enhancing the axonal regeneration.

As illustrated in Figure 1, ASC were stimulated by NGF (NGF-ASC) or VEGF (VEGF-ASC) or without growth factor (ASC) for 72 h and resulting secretome (STM), i.e., STM-NGF-ASC, STM-VEGF-ASC and STM-ASC was subsequently used for DRG assay for 48 h. When the ASC were not stimulated prior to DRG assay, STM derived from the ASC was either supplemented with NGF (STM-ASC+NGF) or VEGF (STM-ASC+VEGF) or no growth factors (STM-ASC) at the time of DRG seeding. As a control, culture conditions with NGF alone (NGF) or VEGF alone (VEGF) or without growth factors (no GF) were used. In all cases, growth factors were applied at 10 ng/mL based on preliminary experiments [34]. For the axonal outgrowth assay, DRG-explants were seeded at a density of one per well onto 24 well plates. Cultures were maintained in a humid atmosphere at 37 °C and 5% CO_2_ for 48 h and images were captured at 5× and 10× magnification. In total, 3 independent experiments resulting in a total of 8 DRG-explant cultures for each experimental condition were performed. 

### 2.6. Immunocytochemistry of DRG Cultures 

After 48 h, DRG cultures were observed under a microscope and bright-field images with phase-contrast were taken at 5× magnification using a Zeiss Axio Vert.A1 inverted fluorescent microscope (Carl Zeiss AG, Jena, Germany) and a Zeiss AxioCam MRc camera (Carl Zeiss AG, Jena, Germany). DRG-explants were then fixed in 4% PFA at room temperature (RT) for 10 min and permeabilized and blocked in PBS containing 0.1% Triton X-100 and 1% BSA (i.e., dilution buffer) for 60 min at RT. For immunocytochemistry, the cultures were incubated overnight with the following primary antibody at 4 °C: monoclonal mouse anti-β-Tubulin III (1:1000, Sigma-Aldrich, Cat. No. T8578) for axons. The cultures were then washed in PBS and incubated with the following secondary antibody: sheep anti-mouse Cy3 (1:500, Sigma Aldrich, Cat. No. C2181) and Hoechst 33258 nuclear staining (1:1000, Sigma Aldrich, Cat. No. 94403) for 60 min at RT. Subsequently, digital images were acquired at 10× magnification (numerical aperture 0.45) by using a Nikon Eclipse Ti inverted fluorescent microscope and a Photometrics prime 95B 25 mm camera. 

### 2.7. Quantitative Measurements of Axonal Outgrowth

DRG-explant cultures were analyzed for axonal length and axonal area in an automated manner with a standardized analysis mask created using the Nikon NIS-Elements AR image analysis software. The DRG area was defined in the Hoechst channel and the intensity threshold was set to be 45. The area occupied by axonal outgrowth was evaluated in the Cy3 channel and the intensity was set to be 65. For accurate axonal area measurements, no binary processing for “fill holes” was selected. For analysis of the axonal length, on the other hand, binary processing for “fill holes” was used. A binary operation expression was used for defining the origin of axonal outgrowth from DRG as well as axonal endpoints. The shortest perpendicular distances from the axonal growth origin to the endpoints were measured and the output was displayed automatically.

### 2.8. Stimulated ASC for Animal Studies

ASC were harvested and cultured as explained earlier. Briefly, ASC were cultured in 75 cm^2^ flasks at a density of 13000 cells/cm^2^ and stimulated by NGF 10 ng/mL for 72 h prior to transplantation.

### 2.9. Fibrin-Hydrogel Nerve Conduits (FNC)

FNC were prepared according to the manufacturer’s instructions as described earlier (Tisseel Kit VH 1.0, Baxter, SA, USA) [42,43]. Briefly, Tisseel provides a fibrinogen solution containing fibrinogen 100 mg/mL, factor XIII 0.6–10 IU/mL, plasminogen 40–120 mg/mL, aprotinin synthetic 3000 KIU/mL, and a thrombin solution 500 IU/mL with calcium chloride 40 μmol/mL. Fibrin conduits measuring 14 mm in length, 1 mm in wall thickness and 2 mm in lumen were produced for bridging a 10-mm nerve gap injury. The thrombin 500 IU/mL was diluted in sterile water to 30 IU/mL. Equal amounts of the diluted thrombin and the fibrinogen solution were mixed directly into a silicone mold pre-set with a stainless steel lumen and incubated for 30 min at 37 °C for polymerization. Resulting FNC constructs were stored in sterile PBS at 4 °C for a maximum of 24 h before animal experiments.

### 2.10. Intramural Delivery of ASC

About 2 million NGF-stimulated ASC or unstimulated ASC were incorporated into the wall of the conduit, the resulting fibrin conduits were designated as FNC-W(NGF-ASC) and FNC-W(ASC) respectively. Immediately before transplantation, cells were trypsinized and suspended in a volume of 540 μL Fibrinogen. Further, 60 μL of thrombin solution 30 IU/mL was added to the ASC-Fibrinogen suspension and the resulting solution was cast into the FNC.

### 2.11. Intraluminal Delivery of ASC

About 2 million NGF-stimulated ASC or unstimulated ASC were incorporated into the lumen of the conduit, the resulting fibrin conduits were designated as FNC-L(NGF-ASC) and FNC-L(ASC) respectively. Cells were suspended in the final volume of 20 μL of Fibrinogen solution (25 mg/mL). Further, 20 μL of thrombin was added to the ASC-Fibrinogen suspension. The resulting solution was injected into the lumen of the FNC and incubated for 30 min at 37 °C for polymerization.

### 2.12. Surgical Procedure and Experimental Groups In Vivo

All the studies were conducted in accordance with the local veterinary commission in Basel, Switzerland (No. 2925). About 12-week old female Sprague-Dawley rats weighing 250–300 g (Janvier, Mayenne, France) were used for the experiment. A total of 36 rats were operated and randomly categorized into 6 groups: (1) autograft, (2) fibrin-hydrogel nerve conduit without cells, i.e., FNC, (3) FNC assisted intramural delivery of unstimulated ASC, i.e., FNC-W(ASC), (4) FNC assisted intramural delivery of NGF-stimulated ASC, i.e., FNC-W(NGF-ASC), (5) FNC assisted intraluminal delivery of unstimulated ASC, i.e., FNC-L(ASC), (6) FNC assisted intraluminal delivery of NGF-stimulated ASC, i.e., FNC-L(NGF-ASC). All surgical procedures were performed under general anesthesia with 3% isoflurane. Routinely, Buprenorphine (Temgesic ®, 0.3 mg/mL, 0.05 mg/kg, Indivior AG, Baar, Switzerland) was administered before and after the surgery. The left sciatic nerve was approached dorsally using a gentle spreading technique of the gluteus muscle. The sciatic nerve was transected 5 mm distal to the gluteal branch and nerve ends were inserted 2 mm into the fibrin conduit and fixed to the conduit by a single epineural suture (9/0 nylon, S&T). Muscles and fascia layers were closed with a single resorbable stitch (5/0 Vicryl, Ethicon) and the skin was closed by a continuous running suture (5/0 Vicryl, Ethicon). For analgesia, the animals received subcutaneous injections of Meloxicam (Metacam ®, 2 mg/mL, Boehringer Ingelheim GmbH, Basel, Switzerland) 2 mg/kg BW postoperatively, and then 1 mg/kg BW every 24 h for two days. Further, Paracetamol (Becetamol ®, 100 mg/mL, Gebro Pharma AG, Liestal, Switzerland) 50mg/kg BW/day was added to the drinking water for 7 days. 

### 2.13. Tissue Processing and Immunohistochemistry

**Figure 2:**
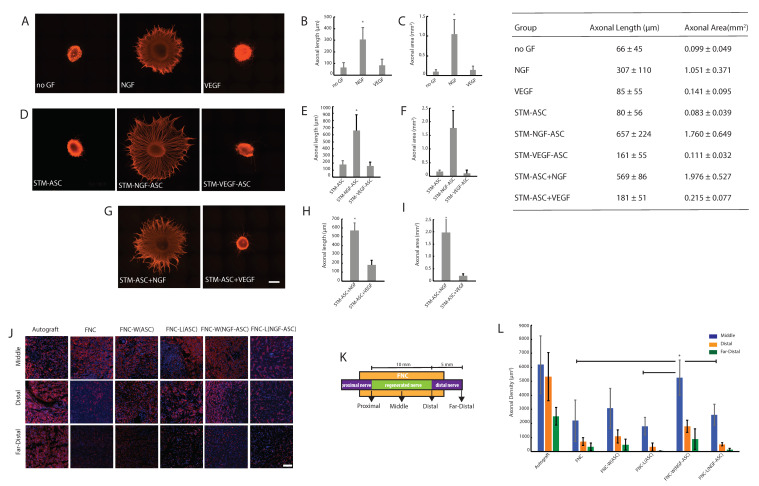
Fibrin assisted delivery of stimulated stem cells for axonal regeneration: (**A**) Microphotographs of DRG-explant cultures treated with growth factors. (**B**) Quantitative measurements of axonal length. (**C**) Quantitative measurements of the axonal area. (**D**) Microphotographs of DRG-explant cultures that were treated with secretome derived from unstimulated ASC (STM-ASC) or NGF-stimulated ASC (STM-NGF-ASC) or VEGF-stimulated ASC (STM-VEGF-ASC). (**E**) Quantitative measurements of axonal length. (**F**) Quantitative measurements of the axonal area. (**G**) Microphotographs of DRG-explant cultures that were treated with ASC’s secretome in combination with exogenous NGF (STM-ASC+NGF) or VEGF (STM-ASC+VEGF). (**H**) Quantitative measurements of axonal length. (**I**) Quantitative measurements of the axonal area. For images (**A**)–(**I**), the scale bar represents 500 μm and the bars represent mean ± SD of n = 8. Significant differences at * *p* < 0.05 are indicated in comparison to all other treatment groups. (**J**) Microphotographs showing the β-Tubulin III+ axons for various experimental groups: Autograft; empty fibrin-hydrogel nerve conduit “FNC”; FNC’s wall loaded with unstimulated ASC, i.e., intramural ASC delivery “FNC-W(ASC)”; FNC’s lumen loaded with unstimulated ASC, i.e., intraluminal ASC delivery “FNC-L(ASC)”; FNC’s wall loaded with NGF-stimulated ASC, i.e., intramural NGF-ASC delivery “FNC-W(NGF-ASC)”; FNC’s lumen loaded with NGF-stimulated ASC, i.e., intraluminal NGF-ASC delivery “FNC-L(NGF-ASC)”. The scale bar represents 100 μm. (**K**) Anatomical segmentation representing the histological analysis of the reconstructed nerves. (**L**) Quantitative measurements of β-Tubulin III+ axons for various experimental groups: Autograft; empty fibrin-hydrogel nerve conduit “FNC”; FNC’s wall loaded with unstimulated ASC, i.e., intramural ASC delivery “FNC-W(ASC)”; FNC’s lumen loaded with unstimulated ASC, i.e., intraluminal ASC delivery “FNC-L(ASC)”; FNC’s wall loaded with NGF-stimulated ASC, i.e., intramural NGF-ASC delivery “FNC-W(NGF-ASC)”; FNC’s lumen loaded with NGF-stimulated ASC, i.e., intraluminal NGF-ASC delivery “FNC-L(NGF-ASC)”. Blue staining is Hoechst indicating cell nuclei. The bars represent mean ± SD of n = 6. Significant differences at * *p* < 0.05 are indicated in comparison to all other experimental groups.

At 4 weeks post-operation, all the animals were euthanized by CO_2_ and regenerated nerve tissue was harvested with the proximal and distal nerve stumps. The harvested nerves were embedded in OCT freezing media (Tissue-Tek, Sakura, Japan) and flash-frozen in liquid nitrogen through 2-methylbutane (Sigma Aldrich, Cat. No. M32631). Nerve cross-sections were prepared by cryostat from the middle, distal and far distal part of the explanted nerve tissue as shown in Figure 2K. The middle sections were taken right in the middle of the explanted conduit, the distal sections at the distal suture point, and the far distal sections 5mm distal to the distal suture. Serial 5 μm thick tissue sections were prepared onto slides (Superfrost plus, Menzel-Gläser, Braunschweig, Germany) and stored at −80 °C. For staining, every second section was processed. First, the slides were fixed in 4% PFA for 10 min and washed in distilled water and then blocked using dilution buffer for 60 min at RT. Slides were then incubated overnight at 4 °C with the following primary antibodies: monoclonal mouse anti-β-Tubulin III (1:1000, Sigma Aldrich, Cat. No. T8578) and polyclonal rabbit anti-S100 (1:100, abcam, Cat. No. ab76729). After rinsing in PBS, secondary sheep anti-mouse antibodies Cy3 (1:500, Sigma Aldrich, Cat. No. C2181), donkey anti-rabbit Alexa Fluor 488 (1:500 abcam, Cat. No. ab150061) and Hoechst 33258 (1:1000, Sigma Aldrich, Cat. No. 94403) were applied for 60 min at RT. The slides were mounted using ProLong Gold (Invitrogen, Cat. No. P36930) and digital images were acquired using a Nikon Eclipse Ti2 inverted fluorescent microscope and a Photometrics prime 95B 25 mm camera at 10× (numerical aperture 0.45), 20× (numerical aperture 0.75) and 40× (numerical aperture 0.95) magnification.

### 2.14. Histological Analysis

Following immunofluorescence staining, digital images of 20× magnification were acquired and used for quantitative analysis of anatomical structures. For measuring the axonal density and area occupied by SC, an automated program was performed using the standardized analysis mask created by Nikon NIS-Elements AR image analysis software. Axonal count and nerve area values were used for calculation of axonal density. Similarly, the area occupied by SC was given in reference to the nerve area.

### 2.15. Statistical Analysis

Data were analyzed by two-way analysis of variance (ANOVA) following Bonferroni procedure with post hoc multiple comparisons using SPSS (version 15.0; SPSS, Chicago, IL, USA). Values with *p* < 0.05 were considered significant. 

## 3. Results

### 3.1. Characterization of Isolated ASC 

ASC were isolated, cultured and resulting cells were characterized phenotypically by immunocytochemistry. ASC were found to be positive for mesenchymal marker CD29 (87%), CD44 (78%), CD90 (81%) and CD105 (85%), and negative for hematopoietic marker CD45 (Figure S1).

### 3.2. Stem Cell Derived Secretome and Axonal Growth In Vitro

Consistent with our previous reports [34], DRG explants exhibited an important and dense axonal outgrowth in response to NGF-stimulation (Figure 2A). Quantitative measurements of axonal outgrowth, i.e., axonal length (in μm) and axonal area (in mm^2^) resulted in 307 ± 110 and 1.05 ± 0.37 (Figure 2B,C). In contrast to NGF, stimulation with VEGF or without growth factors (no GF) resulted in only minimal axonal length, i.e., 85 ± 55 and 66 ± 45 (Figure 2B), which are consistent with axonal area measurements, i.e., 0.14 ± 0.10 and 0.10 ± 0.05 (Figure 2C) respectively. 

Interestingly, STM-NGF-ASC enhanced the significant axonal outgrowth, i.e., 657 ± 224 and 1.76 ± 0.65 (Figure 2D,E). In the case of STM-ASC, no significant axonal outgrowth could be observed, i.e., 80 ± 56 and 0.083 ± 0.039 (Figure 2F). Together these observations clearly indicate the significantly enhanced potency of ASC in response to the NGF-stimulation for promoting axonal regeneration in vitro (Figure 2D–F). 

In contrast to NGF conditions, STM-VEGF-ASC did not result in the enhancement of axonal outgrowth, i.e., 161 ± 55 and 0.111 ± 0.032 (Figure 2E). These observations indicate no significant improvement of ASC’s potency in response to VEGF-stimulation for supporting axonal regeneration in vitro (Figure 2D–F).

In line with STM-NGF-ASC, STM-ASC+NGF culture condition resulted in a robust axonal outgrowth, i.e., 569 ± 86 and 1.98 ± 0.53 (Figure 2G–I). Together these results underline the important role of NGF for promoting axonal regeneration (Figure 2B,E,H). In contrast to these results, the STM-ASC+VEGF culture condition did not elicit synergy as evidenced by axonal outgrowth measurements, i.e., 181 ± 51 and 0.22 ± 0.077 (Figure 2H).

### 3.3. Stimulated Stem Cells and Delivery Route Impacted Early Nerve Regeneration

Considering in vitro findings on the enhanced potency of ASC in response to NGF-stimuli, NGF-ASC were further investigated in rats for treating a 10 mm sciatic nerve gap-injury. The impact of different ways of delivering these cells on the early nerve regeneration, i.e., intramural vs. intraluminal delivery, was also analyzed (Figure 1). 

As depicted in Figure 2J, histological recovery of the treated animals was measured by analyzing the β-Tubulin III+ axons (Figure 2J,L) and S100 + SC structures (Supplementary Figure S2A,B) from the middle, distal and far-distal parts of the regenerated nerve tissue. In the middle part of the fibrin conduit, axonal regeneration (i.e., number of axons/ mm^2^) resulted in the order of 6190 ± 2061, 5260 ± 1257, 3075 ± 1432, 2614 ± 743, 1810 ± 629 and 2197 ± 1478 for autograft, FNC-W(NGF-ASC), FNC-W(ASC), FNC-L(NGF-ASC), FNC-L(ASC) and FNC treatment groups respectively. These results reveal the enhanced axonal regeneration supported by the NGF-stimulated ASC. On the other hand, the superior performance of intramural stem cell delivery was evident compared to the intraluminal delivery route (Figure 2J,L). Together, these results reveal the autograft matching performance of NGF-stimulated ASC in combination with the intramural delivery route i.e., FNC-W(NGF-ASC). Furthermore, the general tendency of enhanced axonal regeneration supported by the FNC-W(NGF-ASC) is evident in distal and far-distal segments in comparison to all other treatment groups (Figure 2J,L), although the differences between some of the groups were not statistically significant (Figure 2L). 

In contrast to the distinct axonal growth response (Figure 2J,L) found for the various stimuli conditions, SC exhibited no differences (Figure S2A,B). However, the molecular differences for the recruited SC in the animals due to the various stimuli conditions remain to be investigated. We hypothesize that the Schwann cells recruited in response to the various stimuli conditions may vary in their phenotype (sensory vs. motor) and secretome.

## 4. Discussion

We hypothesized impactful change in the ability of ASC in response to growth factors’ stimuli for supporting the axonal regeneration. Therefore, the present study was designed to investigate the growth promoting capacity of ASC following NGF- or VEGF-stimulation in vitro and to further translate in vitro findings into animals in combination with two different cell delivery approaches, i.e., FNC assisted intramural vs. intraluminal ASC loading for studying the early nerve regeneration. 

In the present study, experiments were conducted using VEGF or NGF stimulated-ASC. A large portion of the research investigating the neurotrophic effect of ASC involved SCLC derived from ASC while studies on undifferentiated ASC remained scarce [5,44]. Although an SCLC phenotype seems to be desirable for optimal regenerative support, 2 to 4 weeks of in vitro differentiation process represents a major obstacle for the clinical use of differentiated ASC. On the other hand, the therapeutic benefits of differentiated vs. undifferentiated ASC are still not clear. Nevertheless, several studies suggest a similar therapeutic potential for both cell types [29,45,46]. There are two mechanisms explaining the regenerative potency of undifferentiated ASC. The first hypothesis states that ASC might undergo an in vivo trans-differentiation in response to the regenerative microenvironment [21,45,47,48]. The other hypothesis postulates that the neurotrophic potential of ASC may lie in the secretome containing a wide range of biochemical and molecular factors [49]. ASC exosomes releasing miRNA21, miRNA222 and miRNAlet7a play an important role in neuronal survival by inhibiting apoptotic pathways. In addition, the secretome of ASC contains nerve growth factor (NGF), glial cell-derived neurotrophic factor (GDNF), brain-derived neurotrophic factor (BDNF), neurotrophin-3 (NT-3), insulin-like growth factor 1 (IGF-1), vascular endothelial growth factor (VEGF), epidermal growth factor (EGF), basic fibroblast growth factor (bFGF), transforming growth factor beta (TGF-ß), and platelet-derived growth factors (PDGF) [50,51,52,53,54]. Furthermore, transplanted ASC may enhance the recruitment of endogenous SC to the injury site [29]. 

To the factors secreted by ASC, adult neurons may respond differently in contrast to embryonic neurons, as the trophic dependency of the various subsets of the neurons in the peripheral nervous system may change with the age. However, several studies have shown that injury-induced expression of various growth factors and surface receptors that are responsive for both adult and embryonic neurons [34,55]. Thus, the embryonic DRG assay used in our study should provide relevant information on the neurotrophic capacity of the secretome derived from ASC.

The data obtained in the present study clearly showed the enhanced ability of ASC in response to NGF-stimulus for axonal regeneration. The secretome derived from ASC after 72 h of NGF-stimulation (i.e., STM-NGF-ASC), as well as the secretome of unstimulated ASC supplemented with exogenous NGF (i.e., STM-ASC+NGF) induced the significant axonal outgrowth. Further on, there is no significant difference found in the axonal outgrowth promoted by either of these culture conditions indicating the growth-promoting function of NGF in combination with ASC’s secretome. However, the exact mechanism of the underlying molecular functions needs to be determined along with the analysis of the secretome profile of ASC resulting from various experimental stimuli. On the contrary, VEGF culture conditions did not result in enhanced axonal regeneration in vitro. These observations may indicate no direct effect of VEGF in vitro on axonal regeneration. Interestingly, ASC secretome contains a wide range of growth factors, in addition to high amounts of VEGF (i.e., 2000 to 3000 pg/mL) [53,54] indicating that there may be no need for additional exogenous VEGF administration for ASC therapies. Thus, the in vitro results and data instructed the design of animal experiments using single factor NGF stimulated ASC.

In agreement with our in vitro findings, NGF-stimulated ASC exhibited a potential for promoting enhanced early nerve regeneration as evidenced by the increased number of β-Tubulin III+ axons. Functional restoration of human nerve injuries is critical and controlled by timely entry of regenerative axons into the distal nerve segment, which in turn is influenced by the quality (i.e., axonal growth speed) and quantity (axonal number) of early axonal regeneration. Thus, the present study reports on the important aspects of early nerve regeneration in the context of ASC-fibrin-hydrogel based therapeutic conduits.

In the middle of the fibrin conduit, i.e., regenerated nerve tissue, the density of regenerating axons for the animals treated with intramural delivery of NGF-stimulated ASC is statistically comparable to the autograft treatment, indicating not only the benefits of stimulated stem cells but also the importance of the cell delivery route. However, the outcome in the subsequent distal and far-distal segments is significantly higher for the autograft group. Further on intramural delivery of NGF-stimulated ASC, i.e., W(NGF-ASC) promoted better axonal regeneration as evidenced by the increased axonal number in the different parts of the conduit including the far-distal segment, although the axonal growth was found in the distal part of the conduit in all the other groups. The beneficial effects of delivering individual VEGF for nerve regeneration are unclear, given the number of studies reporting with variable outcomes, i.e., increased angiogenesis with or without functional benefits [56,57]. On the other hand, studies reporting in the literature with sequential release of VEGF and NGF exhibited beneficial effects. However, early axonal regeneration in those animals appeared poor than autograft animals as evidenced by significantly lower axonal regeneration in the middle graft after 4 weeks [58]. Therefore, the regeneration levels achieved in our present study appeared superior to that of dual-factor NGF and VEGF treatment, indicating the potential of the NGF stimulated-ASC for better nerve regeneration and for reducing the complexity of administering additional exogenous growth factors. 

In order to improve the outcomes, i.e., suboptimal results obtained particularly at the distal part of the nerve, we would need to further refine our technology by optimizing the loading density of stem cells and by incorporating the topographical guidance structures. Given the focus i.e., early nerve regeneration of the present study, we measured only early axonal regeneration. However, the data and knowledge obtained in the present study create strong rationale for the further evaluation of functional restoration, i.e., behavioral recovery, electrophysiological recovery and re-innervation in a long-term study.

The delivery of cells within a conduit is a subject of research and the most optimal delivery system remains to be determined. Particularly for FNC, only a few methods have been evaluated separately and no systematic comparison was reported so far. In general, fibrin solution loaded with cells can be injected into the lumen of the FNC [27]. An alternative approach is to integrate the fibrin solution loaded with the cells into the main structure of the FNC’s wall by polymerization [26]. The least interesting way is to suspend the cells in a carrier medium and to place them into the lumen of the conduit [30], which usually results in leakage of cells.

In our study, we tested the first two approaches by delivering the NGF-stimulated ASC either by FNC assisted intramural or by intraluminal loading. For this, fibrin conduits measuring 14 mm in length, 1 mm in wall thickness and 2 mm in lumen were produced for bridging a 10-mm nerve gap injury. It is widely accepted that fibrin hydrogels possess macro porous structures with a pore size of 10–20 μm [59]. Therefore, conduits used in our study may possess pore sizes in the range of 2–4 μm and 10–20 μm respectively for intramural and intraluminal fibrin structures that can naturally facilitate the diffusion of ASC secretome. ASC based exosomes and microvesicles are in the range of a sub-micrometer size (i.e., 150 nm to 200 nm) [49,60]. Thus, the macro porous fibrin hydrogels enable easy passage of nanometer-sized vesicles and related soluble factors. However, the density of transplanted cells within the hydrogel microenvironment may account for different outcomes achieved through different delivery routes in the present study. In the case of the intramural delivery system, 2 million cells were loaded in the 600 μL of carrier fibrin hydrogel in contrast to an intraluminal delivery system where 2 million cells were loaded in 40 μL of carrier hydrogel. Although homogeneity of cell treatment (i.e., the dose of the total cells) for animals was ensured, the high density of cells within the lumen of the fibrin nerve conduit may result in the high concentrations of the local growth factors. Thus, the highly enriched luminal microenvironment of the intraluminal delivery system may impede the speed of axonal regeneration in contrast to the intramural delivery system. Taken together, the underlying structural and cellular factors may explain appropriately the better outcome achieved by the intramural cell delivery system in the present study.

Fibrinogen concentrations used in the present study for both intramural and intraluminal constructs were based on our earlier research work and no-detrimental effects were found when the fibrin matrix was used alone. As we previously reported [29], ASC showed survival signals up to 14 days after transplantation through nerve conduit. These findings indicate that the regenerative effects of transplanted ASC are mediated by the initial boost of released growth factors. The cell delivery route dependent outcome observed in our present study may partly be attributed to the potency of the transplanted cells. Further investigation on the spatiotemporal profile of the transplanted cells may provide important understanding and knowledge for the optimization of ASC therapies. The present study was conducted using rat-derived ASC, therefore further studies are required using human ASC, in order to translate these findings into clinical settings.5. Conclusions

We report on a growth factor based strategy for ex-vivo stimulation of ASC and show the evidence for the enhanced neurotrophic potency of ASC in response to NGF-stimulation, but not VEGF, to promote axonal regeneration in vitro and in vivo. Furthermore, our study reveals the importance of a fibrin-hydrogel conduit assisted intramural delivery system in improving the therapeutic efficacy of ASC for nerve regeneration. Together, these findings provide new knowledge and important insights for the development of ASC based therapies for treating nerve gap-injuries. Further studies are required for assessing the long-term impact of the ex-vivo stimulated ASC on anatomical and functional nerve regeneration, and for understanding the secretome profile of the ASC resulting from various stimuli-conditions. 

## Supplementary Materials

The following are available online at https://www.mdpi.com/2306-5354/7/2/42/s1, Figure S1: Stem cell characterization, Figure S2: (A) Immunostaining of Schwann cell growth. (B) Quantitative analysis of Schwann cell growth.
