# Supplementary material for: Ex-Vivo Stimulation of Adipose Stem Cells by Growth Factors and Fibrin-Hydrogel Assisted Delivery Strategies for Treating Nerve Gap-Injuries
Source: Bioengineering (Basel). 2020 May 5;7(2):42. doi: 10.3390/bioengineering7020042 (PMC7357460; doi:10.3390/bioengineering7020042)
Supplement: Supplementary file 1 [file bioengineering-07-00042-s001.pdf]

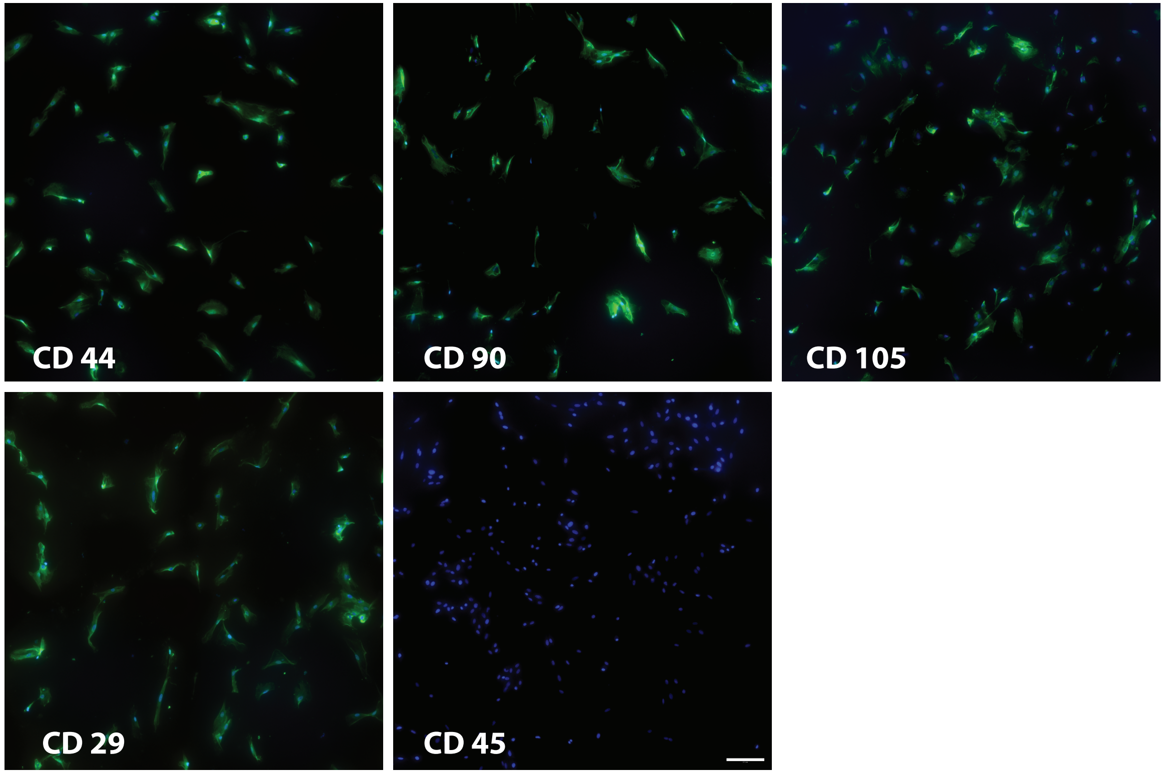

Figure S1. Stem cell characterization. Immunostaining and characterization of rat adipose stem cells (ASC) that are positve for mesenchymal stem cell markers CD44, CD 90, CD105 and CD29 in green. ASC are negative for hematopoietic marker CD45. Blue staining is Hoechst for labeling cell nuclei. The scale bar represents 100 µm.

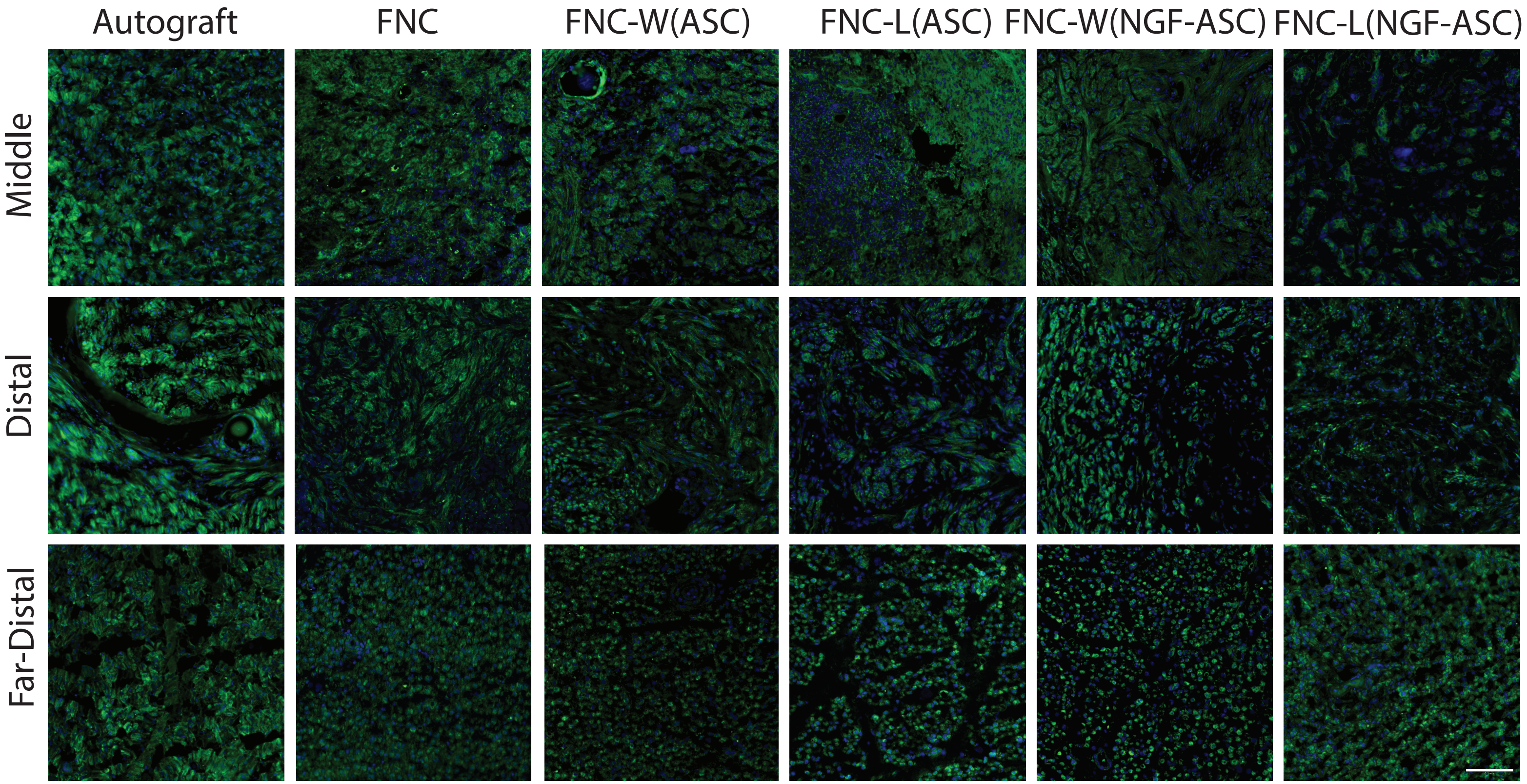

Figure S2A. Immunostaining of Schwann cell growth. Microphotographs showing the S100+ Schwann cell structures for the experimental groups: Autograft; empty fibrin-hydrogel nerve conduit "FNC"; FNC's wall loaded with unstimulated ASC, i.e., intramural ASC delivery "FNC-W(ASC)"; FNC's lumen loaded with unstimulated ASC, i.e., intralumen ASC delivery "FNC-L(ASC)"; FNC's wall loaded with NGF-stimulated ASC, i.e., intramural NGF-ASC delivery "FNC-W(NGF-ASC)" and FNC's lumen loaded with NGF-stimulated ASC, i.e., intraluminal NGF-ASC delivery "FNC-L(NGF-ASC)". Blue color is Hoechst for labeling cell nuclei. The scale bar represents 100 µm.

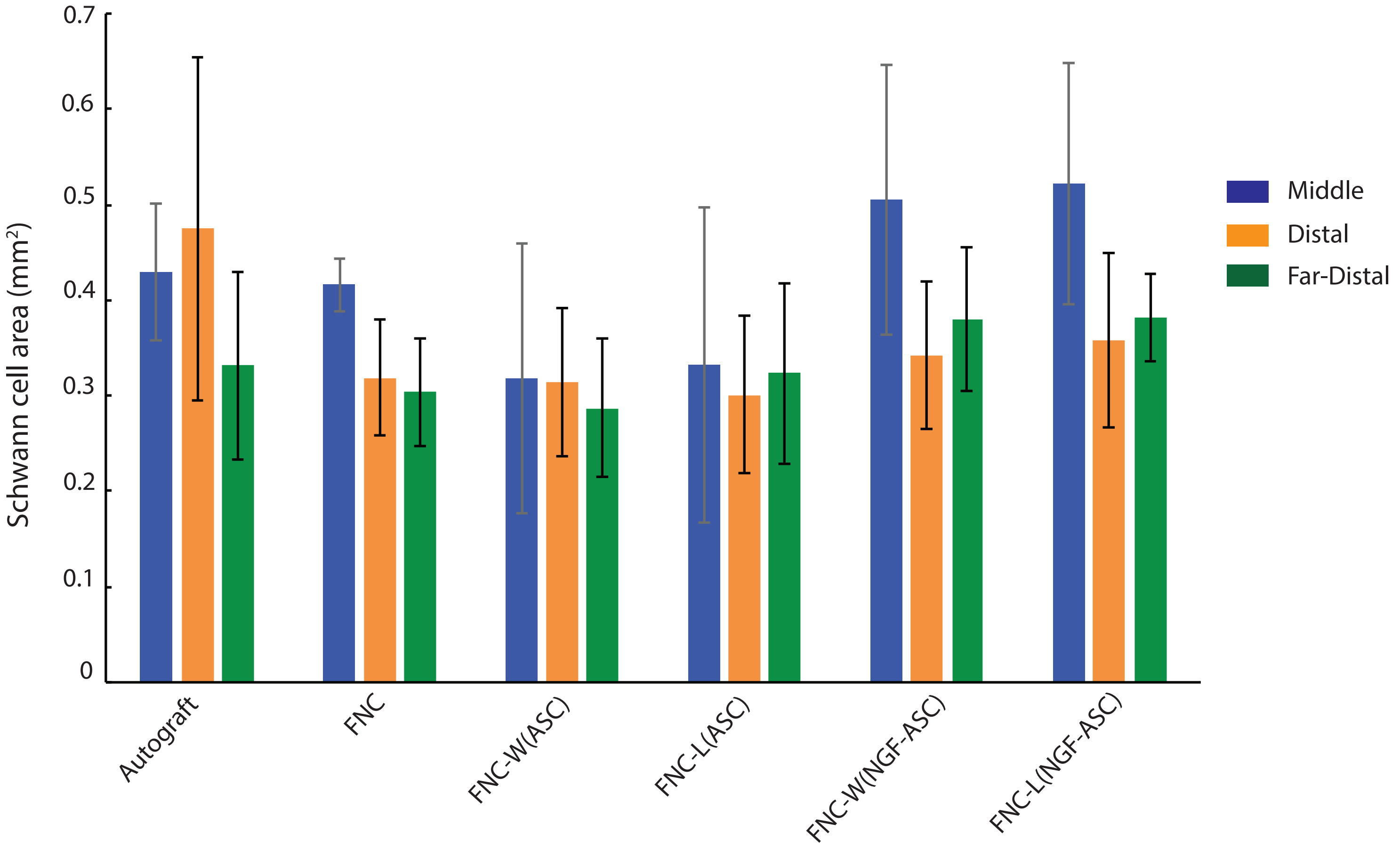

Figure S2B. Quantitative analysis of Schwann cell growth. Measurement of the $S100^+$ Schwann cell structures for the experimental groups: Autograft; empty fibrin-hydrogel nerve conduit "FNC"; FNC's wall loaded with unstimulated ASC, i.e., intramural ASC delivery "FNC-W(ASC)"; FNC's lumen loaded with unstimulated ASC, i.e., intralumen ASC delivery "FNC-L(ASC)"; FNC's wall loaded with NGF-stimulated ASC, i.e., intramural NGF-ASC delivery "FNC-W(NGF-ASC)" and FNC's lumen loaded with NGF-stimulated ASC, i.e., intraluminal NGF-ASC delivery "FNC-L(NGF-ASC)". The bars represent mean ± SD of n=6.
